# When craft kicks back: Embryo culture as knowledge production in the context of the transnational fertility industry

**DOI:** 10.1177/03063127221083869

**Published:** 2022-03-17

**Authors:** Elina Helosvuori, Riikka Homanen

**Affiliations:** Tampere University, Tampere, Finland

**Keywords:** embryo culture, IVF, knowledge production, standardization, craftwork, transnational bioindustry, multi-sited ethnography

## Abstract

The multibillion-dollar fertility industry promotes standardization in *in vitro* fertilization laboratories. Transnational pharmaceutical and biotechnological giants distribute a wide range of fertility products, from embryo culture mediums and incubator technologies to add-ons such as time-lapse embryo monitoring. These technologies are designed to standardize and automate knowledge production regarding embryonic viability. More effective knowledge production enables the more effective selection of embryos for transfer, which in turn leads to more future babies and enables economic scaling-up. Drawing on two multi-sited ethnographic studies at eight fertility clinics in Finland during 2013–2020, this article discusses how knowledge about embryos is produced in the processes and practices of embryo culture. We argue that automation and standardization in clinical practice are not always perceived as economically desirable. Sometimes standard technologies do not replace hands-on knowledge production, although they may transform it. The technologies are also perceived as modifying the object of knowledge itself in undesired or unnecessary ways. In such cases, concerns are raised regarding the best interests of patients, embryos and future babies, who might be better served by masterful laboratory craftwork. We conclude that embryo culture is not only a site of knowledge production – one that aims to make babies and parents through standard and craftwork knowledge practices – but also a site of multiple bio-economies of assisted reproduction, some of which resist automation and standardization.

The multibillion-dollar fertility industry is being reshaped by ongoing expansion, globalization, the rise of private equity investments, the significance of patenting, and increasing consolidation. Consolidation manifests itself in several ways, including the merging of fertility clinics into larger international chains, and the expansion of company portfolios to incorporate every step of the fertility treatment journey and wider ranges of fertility products (see Spar, 2006; Van de Wiel, 2019). For example, major players in the field, such as the pharmaceutical company Merck and the biotechnology company Vitrolife, offer products that range from embryo culture mediums and incubator technologies to add-ons such as time-lapse embryo monitoring/imaging systems (TLS).

The major industry players distribute their products across the globe, including in Finland, where we conducted research at private fertility clinics and clinical *in vitro* fertilization (IVF) laboratories. Van de Wiel (2019) has argued that alongside their ambition to consolidate the whole IVF journey, the pharmaceutical and biotechnology giants aim to promote standardization and automation at IVF clinics (see also Global Fertility Alliance, 2018). In prior literature on the industrialization of biology-based clinical and research practice, standardization, and automation have been seen as preconditions for scaling up and branching out (Franklin, 2013; Meskus, 2018; Timmermans and Epstein, 2010). It would seem that it is in clinics’ economic interest to be able to treat more patients through more standardized and automated processes and practices, which ideally ensure that selected tasks can be performed in the same manner every time, thus saving manual labor, time and money.

In today’s laboratories, however, standard protocols never entirely work, and automation is only possible up to a point. Meskus (2018) describes how biological cell material can ‘resist’ the standardization of protocols by behaving unexpectedly, even when identical practices are performed. Because of enduring uncertainties regarding the limitless capacity of some biological material, including stem cells and embryos, to manifest new aspects and development variations, there is no clear methodology to automate many of the steps of laboratory work (Meskus, 2018: 165–171). It is not only research laboratories (the topic of Meskus’s study) that face this chronic uncertainty. The standardization and automation of work in clinical medical laboratories (e.g. Singleton, 1998; Thompson, 2005; Van de Wiel, 2018) and medical practice more generally (e.g. Berg, 1998; Bowker and Star, 1999; Timmermans and Berg, 1997) have long been described in science and technology studies (STS) as an attempt to stabilize instabilities or, as Hogle (1995) puts it, to standardize the non-standard.

Laboratory work therefore remains a combination of craftwork, high-tech machinery, standards and the labor power of the biological material itself (Franklin, 2013; Franklin and Roberts, 2006). Pressures and measures to produce more efficiently subject human biological material to larger-scale production, and reconfigure the human labor of managing and manipulating that material (Meskus, 2018: 50). When machines and standards take over, this is seen in general as a challenge to the situated, skilled, manual craftwork that is acquired by working with materials over long time periods (Meskus, 2018; Rothman, 2016; Sennett, 2008). Further, new technologies to standardize and automate clinical medical laboratories inevitably entail new forms not only of value production, but also of knowledge production (see Landecker, 2007; Van de Wiel, 2019).

This article considers embryo culture technologies in IVF clinic laboratories as *knowledge production* technologies that ultimately aim to *know* the most viable embryos to achieve successful pregnancies, which is where the clinics’ business lies. We ask what kind of knowledge is produced in these technological practices and how it is produced in embryo culture process and practices. Further, we analyze what those knowledge practices ‘do’ in the context of the transnational reproductive industry complex. The technologies focused on here – incubators (with and without TLS add-ons) and embryo culture mediums – are designed to enable the standardization and automation of this knowledge production in clinical practice. In short, standardization in embryo culture comprises technological protocols that ideally allow more effective embryo selection by knowing viability more effectively.

Embryo culture can indeed be seen as a representation of how biology becomes technology in the era of assisted reproduction and biotechnological change: *In vitro* embryos are enacted through laboratory science and devices, manipulated by the environment inside and outside the incubator, and fed by substances in the mediums in which they nestle rather than in people’s bodies. However, embryos are also *known* through those technologies, and the knowledge is then fed back into laboratory practices, both for the purpose of embryo culture, that is, by picking (what are perceived to be) the most viable embryos for transfer and also for choosing new mediums and incubators from transnational providers.

Prior studies have examined piecemeal embryo culture and IVF technologies such as pre-implantation genetic screening/diagnosis (PGS/PGD) (e.g. Ehrich et al., 2007; Franklin and Roberts, 2006; Pavone and Arias, 2012) and TLS (Van de Wiel, 2017, 2019). Our research builds on these, but looking at the key technologies used in embryo culture together: incubators, TLS and culture mediums. By exploring all the technologies used and not used in the standard form of embryo culture, we are able to observe trajectories of embryo culture today.

This article considers embryo culture knowledge-producing technologies not merely as case studies, but as approaches to the broader bioindustry of reproduction, as the context where new innovations emerge and are applied. Our detailed analysis of everyday culturing practices and processes show novel combinations of craftwork and standardization in laboratories in general and clinical laboratories in particular. As we shall show, the industrialization of clinical medical practice and its potential consequences on the IVF-born human children and persons are at stake.

It is possible to observe and even predict embryo morphology, embryogenesis and embryo epigenetics through embryo culture technologies. These possibilities are affected by global and local economic pressures to standardize and automate as well as *not* to standardize and automate. We argue that automation and standardization in clinical practice are not always perceived as economically desirable; nor do they threaten skilled, hands-on and experience-based local practice, although they may somewhat transform it. Sometimes the knowledge possibilities offered by standard technologies are seen not as replacing or standardizing hands-on knowledge production, but as modifying it along with the object of knowledge itself in undesired or unnecessary ways. In such cases, concerns are raised regarding the best interests of patients, embryos and future babies who might be better served by masterful laboratory craftwork. To loosely apply Barad’s (2007: 214–215) notion that the world refuses to behave in the ways we intend: Sometimes craft kicks back. We conclude that embryo culture is not only a site of knowledge production – one that aims to make babies and parents through standard and craftwork knowledge practices – but also a site of multiple economies of assisted reproduction, some of which resist automation and standardization.

## Practices of knowing in the laboratory

Theoretically, our article draws on the STS insight that knowledge both describes and constitutes its objects (e.g. Jasanoff, 2004; Knorr-Cetina, 1981; Latour and Woolgar, 1986). Accordingly, we conceptualize embryo culture as ways of knowing about embryonic life and viability. We aim to explore technological processes and practices of knowledge production and how they produce (what is perceived to be) legitimate and plausible knowledge about embryos and their viability.

Knowledge production in the laboratory is generally seen as ‘through and through relational, embodied, affective and practical’ craftwork (Meskus, 2018: 108). It is work that involves a multitude of actors, local and global, human and non-human. In IVF laboratories those actors include technologies, standards, embryologists, other clinical staff, intended parents, national and supranational laws and regulations on the medical use of human tissues and cells, the industrial players and the living biomaterial itself (see Helosvuori, 2019). It is hands-on work, but ‘there can be no separation of the hands-on and the intellectual reshaping of what cells are [and] what they can do’ (Landecker, 2007: 26), as shown by pioneering laboratory studies (e.g. Knorr-Cetina, 1981; Latour and Woolgar, 1986; Lynch, 1985). As Sennett (2008) describes, craft is characterized by affective and attentive engagement with its object material to fulfill ‘the desire to do a job well for its own sake’.

However, craftwork is subject to increasing political and economic demands for its transformation into more standardized and automated systems of knowledge production, moving from small-scale craft-like surroundings to industrialized settings – scaling up. While standards and protocols may be more common in clinical work than in experimental research, they also become appropriated for uses for which they were never imagined in their design (Bowker and Star, 1999; de Laet and Mol, 2000), used in reflexive rather than clear-cut ways, worked around (Timmermans, 2015), and tinkered with to make them *work* (de Laet and Mol, 2000; Timmermans and Epstein, 2010).

In this article, we introduce a response to standardization that does not rest on hype, hope or excitement. Some clinics resist standardization by reinstating aspects of the craft production of knowledge, in the process sketching new political realities. This response has previously been identified in work practices such as artisanal food production, the home birth movement (Rothman, 2016) and even research management (Davies and Horst, 2015), but it has not been identified in laboratory work to date.

STS approaches have historically been accused of being apolitical or siding with the strong due to their inability to fully theorize how and to what ends materials link to the capitalist mode of production or institutional power relations (e.g. Braunmühl, 2018; Law, 2004: 13–14; Mol and Mesman, 1996; Rekret, 2018). It can be argued, however, that STS approaches to knowledge production and its methods provide the tools to attend to ‘goodness’ in various activities: caring for many participants simultaneously including oneself (see Davies and Horst, 2015; Homanen, 2019; Lemke, 2018: 45; Mol, 2008) and encompassing all participants’ economic conditions. This caring exists alongside the logics and interests of economic value accumulation and instrumentalization.

## Data and methods

To incorporate various laboratory knowledge production practices across different locations, we use multi-sited ethnography (Falzon, 2009; Hine, 2007). Multi-sited ethnography recognizes that the research’s matter of concern is constantly crafted in a range of locations and practices, including laboratories, doctors’ offices, intended parents’ private reproductive voyages and peer support groups. An ethnographic time frame is needed to deal with the entire trajectories of embryo culture.

The article is based on two multi-sited ethnographic studies conducted at a total of eight different private fertility clinics in Finland during 2013–2017, with a follow-up study from autumn 2019 onward. Both of our larger research projects are concerned with the constitution of social relations and selves in clinical reproductive healthcare practices in the context of healthcare marketization. The fieldwork sites encompass fertility clinic spaces including IVF laboratories, conferences and events and peer support groups for intended parents. During our fieldwork periods, there were between ten and fourteen commercial fertility clinics in Finland in total. The combination of our data not only allows us to analyze material from eight of those commercial clinics, but also to observe the links, variations and tensions among practices that would not have been visible in our respective separate data sets.

As is common in ethnographic enquiry, we repeatedly reframed our analysis of our material through new knowledge produced collaboratively with participants in the field (Hammersley and Atkinson, 1995; Holmes and Marcus, 2008). This enabled us to examine how knowledge practices are realized and challenged in specific, situational, everyday clinical work. It also enabled us to see the political work involved in normalizing technologies and in contesting, reformulating and reinforcing technological practices and the market.

Altogether, the data collected in the two larger studies during 2013–2017 and in the follow-up study that started in autumn 2019, comprises ethnographic observations and video recordings (105 videos of appointments and procedures) from six private fertility clinics (clinics A, B, C, D, E, and F) and 27 in-depth interviews with medical personnel in seven clinics (eight clinics participated in total). The videotapes analyzed in-depth in this article comprise recordings of 42 procedures with embryologists in the operating theater and five videos where embryologists are showing and explaining the development of patients’ embryos to them via TLS. The interviews analyzed comprise nine interviews with embryologists and six with doctors, as they are the ones in charge of laboratory work in different ways. The fieldwork was conducted in periods lasting between a few days and two weeks alongside an intense period of participant observation at one laboratory for one month. Additionally, the fieldwork included participant observation at five Finnish and international conferences for medical/laboratory staff, five public events that brought together various stakeholders on (in)fertility issues and two information/recruitment events for potential egg donors organized by private clinics. We also collected documentary material, such as legislative and regulatory documents, care and laboratory protocols and guidelines and hand-outs distributed to intended parents.

Although this article focuses on observations and interviews conducted with specific medical staff in clinics, we also draw on data gathered from observations conducted over seven months in four peer support groups for people experiencing involuntary childlessness, where necessary. A Finnish infertility association organized these groups. Thirteen interviews with intended parents were also conducted.

All interviewees and participants in video recordings signed an informed consent form. Other participants gave oral consent to participate or were informed beforehand of the presence of the researcher and given an opportunity to object or withdraw from the event. At public events and conferences, the researchers informed the organizers of the study beforehand.

## The fertility sector in Finland

The legislation regulating infertility treatment in Finland dates to 2006, when the Act on Assisted Fertility Treatments (1237/2006) was passed. Previously, treatments had been self-regulated by professionals and their associations. The law legalized gamete donation and fertility treatment for single women and (in effect) lesbian couples, but it criminalized surrogacy and banned anonymous donation and any remuneration of gamete donors. A state donor identity register with an identity release system was established. All gamete and embryo donors since 2007 have been registered, and children born as a result of donor IVF may on request receive identity information about the donor after the age of eighteen. Finland has been described as rather permissive (Eriksson, 2017). Indeed, it is so compared with some other European countries, such as Norway and Germany, where egg donation is forbidden.

IVF laboratories are further regulated by the Act on the Medical Use of Human Organs, Tissues and Cells (101/2001), which has been extensively amended since 2007 to align it with common European Union (EU) regulations regarding the procurement, storage, transport and traceability of tissues and cells, and their screening for infection. These regulations were introduced by the EU in the Tissue and Cell Directives of 2004 and 2006 (2004/23/EC; 2006/17/EC; 2006/86/EC). In effect, the EU legislation harmonizes procedures in IVF laboratories across Europe. However, there is no EU legislation on assisted reproduction in general and the overall picture in Europe has been described as a patchwork (ESHRE, 2017).

According to the Tissue Act (101/2001) amended in 2007, procurement, testing, processing, preservation, storage and distribution must take place in a licensed tissue establishment that is registered and regularly inspected by Finnish Medicines Agency (Fimea), a government agency. Additionally, tissue establishments provide Fimea with annual reports. In general (and in line with EU legislation), the Finnish legal requirements include a push toward standardized auditing and quality systems in order to minimize risks to human health (see European Commission, 2016). For traceability purposes, establishments such as IVF laboratories must keep registers of all pharmaceutical products and tissues used, the donors/patients to whom the tissue belongs and all procedures conducted. Furthermore, a register of adverse effects and hazardous situations needs to be kept. Many common technical requirements for the coding, processing, preservation, storage, and distribution of human tissues and cells are also documented in the EU directives and correspondingly referred to in Finnish law.

Finland combines a state-funded Nordic welfare system with a growing commercial care sector. The policy discourse of the centrality of economic competitiveness has been particularly strong in Finland since the 1990s (Mulinari et al., 2009): Public-private partnerships play a vital role in service provision. The Finnish government even works in conjunction with the private sector to attract medical tourists; FinlandCare, a mediator organization coordinating the sale of care services abroad, was established as a collaboration between government agencies and private companies. Compared with Sweden, for example, Finland now has a wide network of private hospitals and clinics. Finland has also become a destination for fertility travel for donor eggs, mainly from other Nordic countries where laws, policies or service systems are more restrictive (for a more detailed description of the transnational Finnish egg donation context, see Homanen, 2018).

During our fieldwork, Finland has had 17–25 clinics with licenses to perform IVF treatments. Of these, 10–14 have been commercial establishments. Over time, private enterprise has moved from maverick small businesses to a few large chains of clinics with links to international investors and branches in Russia and some Baltic countries. The consolidation of the sector in Finland can be seen in ongoing mergers and acquisitions: In a little over 20 years, all but a couple of clinics have been merged with or acquired by another clinic, a chain of clinics or a large healthcare company.

While clinics in Finland thus seem to follow recent global trends in the industry, in many ways they cannot be compared to any of the truly major players in the field. They do not rank among the large, aggressively expanding fertility businesses that operate dozens of clinics and laboratories worldwide: The largest chains in Finland have only three or four clinics; United States market leader IntegraMed Fertility, for example, operates 93 clinics across North America, while the Australian Virtus Health runs its 46 clinics not just in Australia, but also in Ireland, Denmark and Singapore. The Finnish clinics do not prominently advertise their services internationally, nor are donors or large number of donor gametes imported from abroad, and donors are not recruited in larger numbers by ‘overpayments’. Finnish clinics even offer ‘one-stop’ services, unlike companies that break down the IVF treatment process into stages and then outsource some (or all) of those stages to countries with favorable regulations and costs. If one wishes to become a big transnational fertility service provider, it seems one has to engage in such market activities sooner or later.

## The trajectory of embryo culture

In an IVF lab, embryo culture follows the placing of an egg and sperm into a petri dish for fertilization. After fertilization takes place through either IVF or intracytoplasmic sperm injection, the resulting embryos are cultured on petri dishes in one or two different artificial culture mediums until they reach the so-called cleavage stage (days two to three, called the ‘short culture’ in our fieldwork clinics) or blastocyst stage (days five to six, called the ‘long culture’). After this, a single embryo is transferred to the intended mother’s womb. It is a rule of conduct among practitioners in Finland, seen in the treatment protocols at many of the clinics, that only one or (in rare cases) two embryos can be transferred simultaneously. The remaining viable embryos are frozen for possible subsequent treatment cycles. Sometimes embryos to be used for frozen transfer are also cultured after they have been thawed, to ensure that the embryos are alive and start dividing after being frozen.

During the culture period, the embryo petri dishes are kept in artificial incubators that mimic the reproductive environment. Traditionally, embryos are removed from the incubator for assessment, under a microscope, of their quality and stage of development. Instead, the TLS used in some clinics (often simultaneously with more traditional, hands-on assessment methods) can take images of embryos at frequent time intervals, which enables their assessment without removal from the incubator. A TLS can also apply (data-driven) algorithmic software that assists the embryologist to select the best-quality embryo for transfer, potentially improving the chance of a live birth.

## Incubators as knowledge-producing devices: Knowing morphology and embryogenesis

During our observations, IVF success was often reduced to the issue of embryo quality in discussions at clinics and public events. First and foremost, the incubator environments were seen as instrumental to ensuring the maintenance of embryo quality and even to improving it. This was also discussed at peer support group meetings. Patients wondered whether their clinics used the best possible incubators, wanted to know about the expenses and whether they could afford the incubator technologies.

The incubator technologies used at clinics during our fieldwork included a more traditional incubator from which embryos were removed for assessment on a daily basis, as well as a few different TLS (Vitrolife’s EmbryoScope and Primo Vision, Auxogyn’s Eeva, and Esco Medical’s MIRI). The medical staff believed that embryo quality could be improved with TLS because the examination was conducted without removing the embryos from the incubator. TLS do not otherwise improve embryo quality per se, although some research (e.g. Chen et al., 2013; Meseguer et al., 2012) suggests that TLS aid the selection of the best-quality embryo for transfer because the embryos are ‘truly’ known through this technology. Indeed, there is quite a lot of fuss about TLS in both biomedical research and clinical practice. It has been hailed as the most significant and groundbreaking new technology in IVF in decades (see Van de Wiel, 2017, 2018, 2019). Its advocates even believe that TLS will eventually make it possible to recognize chromosomal or genetic deviations in embryos – an art that currently requires a biopsy analysis during PGD/PGS (field notes, public lecture at fertility clinic; see also Daughtry and Chavez, 2018).

The fuss is apparent in the following snapshot from our field notes from the 2013 Nordic IVF Laboratory Society conference where a TLS advocate is describing the technology:

It’s the first day of the Nordic IVF Laboratory Society conference and a presentation about the arrival of time-lapse technology is on. The speaker is an embryologist and is connected to a company that is marketing an application of the device; she hence declares herself to be a bit ‘biased’ in that regard. At the beginning of the presentation, she shows a colourful cartoon drawing in which embryos drink, smoke and party all night while the biologists are out of the lab. The cartoon shows how the badly behaving embryos hear a biologist returning to work and pretend to be well-behaved while she has a look at them. After showing the cartoon, the presenter declares pointedly that ‘with time-lapse, the party is over’: Time-lapse technology enables staff to check on video to see how the embryos were behaving while they were not being monitored. This is important because when left alone, embryos ‘do funny things’. They are ‘naughty’ and ‘deceitful’. The speaker explains that with time-lapse ‘we can see how they behave’. Only those that have behaved in a certain way ‘do become babies’ – the naughty ones ‘will not become babies’.

The advocate implies that the rationale for using TLS is that it produces a *different kind of* knowledge about embryogenesis. TLS offers embryologists more visual and temporal information about embryo development during the culture process, as the system constantly monitors the embryo by taking photos every five to twenty minutes. The resulting videos allow the embryologist to observe and record developmental markers, such as the timing of cell division and the movements of embryo growth. As embryologists in our fieldwork clinics told us, they conventionally take ‘a quick glance’ manually through the microscope once a day to visually examine the embryo’s static morphological appearance – embryos should not be out of the incubators for more than two or three minutes, or their pH levels will drop. TLS knowledge about embryogenesis, reconstructed by its temporality, may ultimately ‘rewrite the facts of life’, as we discussed with a conference exhibitor after the presentation described above. Indeed, one might speak of an epistemic break in the practices of knowing embryos and their viability (in culture, *in vitro*), as the previously invisible temporal dimension becomes the way of knowing and reproductive decision-making (see Van de Wiel, 2017, 2018, 2019).

TLS provide a representation of embryo time which can be manipulated to give visual access to reproductive processes that were previously too slow to be observed. Van de Wiel (2018: 21) argues that TLS introduce a ‘cinematographic turn’ in IVF clinics, making embryogenesis visible as a process for a varied audience. TLS-based information, videos and images are shared routinely with intended parents, allowing patients to visually take part in the scientific work (Landecker, 2007: 123). As Helosvuori (2019) has shown, when embryos are observed through the microscope in the traditional way, intended parents are also provided with information about embryo development and given a role in decisions on embryo selection. In such cases, parents are only *told* about embryogenesis and morphology or shown *static* pictures of (their) embryos. Nevertheless, through such information-sharing, patients become enrolled into the process of knowledge production and even into interventions that render previous conceptions of embryo viability inaccurate. This happens with ‘pity transfers’, where embryos that are (perceived to be) inviable are transferred because the patient wishes it: These transfers sometimes result in the birth of healthy babies, which in turn leads embryologists to revise their views on embryo viability (Helosvuori, 2019).

As we hinted at the beginning of this section, patients are aware of TLS and its additional costs per treatment cycle. It is also one of the few laboratory technologies directly marketed to patients (Pottage, 2018). Thus, TLS involves additional clinical and financial decision-making on the patients’ part.

In the following snapshot from a video recording of a clinic appointment, the embryologist is showing the intended parents an accelerated video of the cultured embryos collated together on-screen:

The embryologist and the intended parent couple are all sitting in front of a big computer screen. The intended mother gives few faint shrieks as the video goes on and points excitedly at the evolving embryo images. The embryologist is simultaneously explaining about the two embryos chosen for transfer on the same day: ‘We could take all these fertilized oocytes and look at them all together. So let’s start [the video] again. Okay. Let’s follow them [the embryos] here. The first thing that we are checking was these pronuclei here. So, the division with the oocyte is okay and the normal pronucleus amount [too]. Very important. And now the next thing that we are looking at in these embryos, which is a good sign for an embryo, when it’s divided on the same day [as] fertilization, and that was on Saturday, when you were [at the clinic for the egg-harvesting and sperm sample] in the morning. So the [same] afternoon, around 25, 26, 27 hours after fertilization, the oocytes start to, the pronucleus starts to disappear, like this and then it divides first time [into a two-cell embryo]. So this type of embryo is, it’s so-called early [cleavage] embryo. And it’s always when … There has been studies of many embryos in the literature, so it’s always a good sign for an embryo when it divides like this. So, I think now this is going as it should be, divides into two. Here. This is a little bit slower embryo. … A little bit slower than this one, but dividing very nicely. [Another one of the embryos chosen for transfer is a] little bit quicker all the time than this one and now this one follows here, now it’s here, but still with the right timing and, going to, you can see this. It’s going nicely. So, there it is at eight cells and here is eight cells, which is a really, the right amount of cells in the embryo on day three’. They talk a bit about the third embryo, which will be cultured until the blastocyst stage and then possibly vitrified. Then the embryologist asks if the couple would like prints of the embryo images. The couple happily accept these and the embryologist says: ‘I will send it to you. This [too], when we see if the embryo goes for freezing. So, after that I will send it to you. But then you had a picture from [a baby], going from, so you can see the kind of embryos that have been transferred. So you will, I will send it in the email’. (video recording, clinic C)

Note that TLS seems to fulfill both an affective and a clinical diagnostic function here. The embryologist straightforwardly juxtaposes pictures of viable embryos with baby pictures, not unlike ultrasound images and videos. As with ultrasound, prenatal life in TLS imaging videos is known without the embodied, experience-based knowledge of the intended mother (see e.g. Duden, 1993; Homanen, 2013). However, unlike ultrasound, TLS places the prenatal life concretely outside the maternal body and rewrites the human origin story accordingly (Van de Wiel, 2017, 2018). Nevertheless, encountering prenatal life – seeing ‘the baby’ for the first time and getting a first picture – is the obvious attraction for the intended parents.

The embryologist in the snapshot also seems to be promoting the notion that there is a universal regularity in the temporal process of embryo development that can be both observed and operationalized to *predict* viability (see Waldby, 2019). Here, she refers to studies of embryo development, but TLS also have a data-driven component. This component allows visual information about temporal and morphological features (which are sometimes invisible to the human eye) to be measured, quantified and analyzed through algorithms to predict embryo viability.

The algorithms are based on historical data sets: The timing of cellular divisions is viewed in light of historical embryo populations to predict success rates, that is, the likelihood that the treatment will result in pregnancy. Clinics are also encouraged to provide their own data for the further development of the algorithmic tools. In principle, then, TLS allow clinics to adopt a quantified, automated, standardized, data-driven method for knowing about viability and selecting embryos through prediction (see Van de Wiel, 2018, 2019).

Where TLS run automated algorithms to predict the viability of embryos cultured at a clinic, one should ideally be able to use this to scale up production by standardizing one’s knowledge production. Data-based prediction is marketed as making it possible to transfer embryos at an earlier stage than would normally be the case, allowing clinics to accommodate more patients with a better success rate.

During our fieldwork, not all clinics took full or even any advantage of this data-driven component, and many embryologists relied at least partially on manual appraisals of embryos, either under the microscope or by rewinding TLS videos backwards and forwards on the computer screen. Embryologists described how one develops ‘an eye’ and ‘a feel’ for embryos after observing them for a long time. This experience-based, hands-on method of examining embryos (i.e. craft know-how) was called ‘a natural way to rule out embryos’ by one of our informants. Some, however, downplayed their experience-based observational skills in embryo examination, regarding it as pointless in comparison with standard quantified information on embryos. This shows that embryologists work at the crossroads of standardized and tacit modes of knowledge production, and that technologies can be used in non-standard and non-automated ways.

Despite TLS’s acclaimed potential to produce valuable information about embryos, its translation into clinical use is not clear-cut. To take full advantage of the technology, staff training and a lot of extra time in the laboratory would be required. ‘One could spend half a day tinkering with them [TLS]’, laughed one embryologist in our study. On the other hand, the meaning of all the markers of embryo development on which TLS reports is not fully known. For instance, we were told that in light of historical data sets, it is believed that cells should divide in a ‘disjunct manner’ so that the cell ‘is not doing something all the time’. However, when we asked further about the meaning of this, we were told that there is no knowledge about what it specifically means for cell quality, let alone for further embryo development.

## Predictive technologies versus masterful laboratory craftwork

Knowing developmental time in the IVF process (through predictive TLS in incubators or by manual e/valuation) affects the decisions on the duration of embryo culture in incubators: For how many days after fertilization should the lab culture embryos that are candidates for transferral to an intended mother’s uterus? There seems to be no strong consensus among clinicians about which transfer stage results in more pregnancies or about the health implications of this for IVF-born children. Clinics mostly follow their own experience-based assessments and small-scale statistics and practice accordingly. Thus, the duration of embryo culture varies, leading to different stages of embryogenesis at transfer.

The embryologist places the eggs and sperm on a petri dish on the day of ovarian pick-up (OPU), that is, the day when eggs are harvested from the ovaries. Two preliminary nuclei (one from the egg and one from the sperm) signify fertilization one day after OPU (day one). Markers indicate whether the cells have begun cleavage (i.e. to form an embryo) two days after OPU (day two). There are six to twelve cells on day three; the morula stage is reached on day four. A blastocyst forms ‘roughly on time’ by days five to six.

The main stages at which embryo transfer is performed are the cleavage stage (days two to three after fertilization) and the blastocyst stage (days five, six or even seven after fertilization). Some of our field clinics advocated the ‘long culture’ process of five to six days and some preferred the ‘short culture’ of two to three days. Some even changed their preferences and practices during our fieldwork period. According to our informants, public university hospitals routinely used the short culture to accommodate more patients and save resources.

In our fieldwork clinics, the short culture was argued for in terms of economy and the superiority of the ‘natural’ womb environment. The economic arguments referred not to the cost-effectiveness of treating as many patients as possible, as fast as possible, with the fewest resources (even though this might also have been taking place), but rather to being economical in the face of the chronic uncertainty of prediction that characterizes the IVF treatment sector. One never knows if embryos that look viable on days one to three will survive until the blastocyst phase. Especially with poor-quality embryos, medical staff were keen on cleavage stage transfer ‘so that there is at least something to transfer’, as one embryologist put it. These arguments were based on the belief that the womb environment is always better for embryo development than the artificial incubator and growth medium, placing hope in the natural(ized) *in vivo* environment to enable viability. This belief was further underpinned by the uncertainty regarding whether embryos that are judged to be of good quality (according to morphology and developmental biomarkers) really implant more often than bad-quality embryos (see Thompson, 2005: 114). Some professionals at laboratories with TLS also relied on the system’s predictive analysis and standardized use: What need is there for the long culture if one can predict longer-term viability?

The prediction of viability is also questioned by professionals. Sometimes markers that appear to be unpromising turn out to be meaningless in a long culture, again because of uncertainty regarding the meaning and relevance of all the stages in development and morphological characteristics. This is apparent in the following interview extract:

Q: Could you explain once more what you observe from the embryos?

A: So we observe in day two and three the cell quantity, … how much fragmentation there is [a sort of graininess on the embryo] and then we have a look at the cells, whether they are the same or different size, it’s better if they are similar size. … Then we have a look at the nuclei, is there a nucleus in every cell. If there are multiple nuclei, that is a bad situation, then there has been something wrong in the cleavage.

Q: But that can be fixed later on, is that right?

A: Yes, that can be fixed. We used to be much more critical toward multiple nuclei and so we dumped those always, mostly, when we saw even one cell with multiple nuclei. But now we have noticed that when they are transferred, it can get fixed, so it is not such a critical factor after all. (embryologist 1, clinic A)

Embryos may develop and implant normally further along the line. There are also genome abnormalities that cannot be predicted in early-term cultures. Recurrent early miscarriage, for example, may be a sign of problems with the sperm genome: It is believed that the genome in the sperm is only activated in the embryo after day three. Problems will therefore not manifest themselves during the cleavage stage and cannot be predicted without PGS/PGD.

Both of the above cases are also good examples of problems not just with prediction in general, but with TLS prediction in particular. TLS analyses are not (yet) sensitive to most chromosomal abnormalities, nor do they take new or local conditions into account very well. This is because they are based on *historical* data sets and not all clinics enter their own embryo population data into the data pool (Van de Wiel, 2018).

Moreover, in contrast to the hype around TLS, some clinics have almost stopped using it. Long culture advocates at some of our fieldwork clinics told us that they did not need the (far-from-foolproof) predictive component of TLS because it did not really benefit clinical IVF outcomes unless one was set on doing only short cultures. Here, TLS was described as ‘merely a good incubator’ and thus as too expensive for clients.

The head doctor explained to me in his office: ‘It is of course interesting to see the temporal development [on-screen] and find out exactly how the cell can fix itself, but it does not matter [that much] clinically what the journey has been like until blastocyst if it looks pretty. … When you master the long culture [manually] in the laboratory, you don’t need TLS anymore. It is only useful when you don’t have the skill to keep the embryos alive [until blastocyst]. … This idea of the womb always being like a warm embrace for the embryo is just not true because the womb is better on day five or six’ [than days two to three, referring to the endometrium being more receptive at that stage of the IVF cycle]. He then went on to elaborate on how there are ways to test the stage of the endometrium to define the exact individual implementation window for the transfer and how that combined with PGS/PGD is the way to IVF success. (head doctor, clinic F)

This head doctor sketches a wholly different picture of TLS as a clinical tool compared with industry advocates or with what the technology’s worldwide sales figures might suggest (e.g. Van de Wiel, 2019). Instead of celebrating detailed knowledge of embryogenesis for clinical purposes, he is of the opinion that TLS is rendered redundant by skilled laboratory work learned through repeated performance over time. He does not share the conception that embryo transfer in the blastocyst phase can be justified with arguments about the caring *in vivo* uterine environment because the optimal time for implantation is understood to be around days five to six, with some individual variation.

Long culture is also perceived as economic, but in somewhat opposite ways to short culture. When long culture is mastered, it is believed, viable embryos are likely to survive. This saves money and labor for all:

It really is a lot more cost-effective, in a way, if we continue [to culture] to day five because many times it can occur that in day two, there are a lot of embryos, and then you transfer one of them into the uterus, and many into the freezer, and then you transfer those day two embryos [later]. It can happen that the patient undergoes multiple transfers with frozen embryos and not with great odds. However, if you continue to day five, you sort of weed out useless [non-viable] embryos … and then the odds per transfer are much higher. (doctor 2, clinic A)

‘Cost-effectiveness’ refers to both patients’ and clinics’ interests. The professionals explained to us that transferring as many embryos as possible with the least effort is not good for business at the end of the day. The pregnancy results remain low and freezing multiple embryos also takes time and effort. Patients are burdened with repeated disappointments and unnecessary medical interventions. It is more lucrative to culture for longer and to transfer and freeze less. According to this industry logic, good news will eventually travel, meaning that in addition to better clinical IVF outcomes, business outcomes improve too.

The business logic, then, does not exclude care for patients or cells, but requires it. Patients’ wishes and practical everyday lives affect the culture duration in other ways as well. Craft is about practicalities (e.g. Meskus, 2018). Cleavage stage transfers may be made for patients who want their transfer to be conducted by a particular doctor who can only perform the transfer on the second day of embryo culture. National holidays are often accommodated. Furthermore, if a patient is eager to ‘get to the point of embryo transfer’ – to ‘push the panic button’, as put in the words of one informant (doctor 2, clinic A) – rather than waiting a couple of days and incurring the risk that the embryos might perish by days five to six, a transfer is made to please them.

Overall, TLS are designed to enable an automated, standard way of knowing embryo viability in terms of embryogenesis and morphology and are thus designed to enable the scaling-up of production. As with standards and standard technologies more generally, they are subject to local adjustment and manipulation in practice (Timmermans and Epstein, 2010). The unpredictability, uncertainty, locality and individuality of embryo viability make it hard for the TLS standard to work and for clinics to capitalize on it. Furthermore, there are practices that resist this automation and standardization for its own sake, for the sake of the intended parents’ lives and finances as well as for those of the clinics. Here, craft has both economic and ethical value.

## The culture growth medium as a technology of (not) knowing epigenetics

The artificial embryo growth medium in which the fertilized ovum is immersed during culture contains mostly glucose, pyruvate and energy-providing components. It is also possible to add amino acids, nucleotides, vitamins and cholesterol to improve embryo growth and development. All in all, the mediums aim to mimic the optimal reproductive environment for each developmental stage (see Landecker, 2016).

However, the big pharmaceutical companies that provide the mediums do not share all the details, such as add-ons or percentages of different chemical ingredients in their products, despite pressure from medical practitioners. For instance, according to our informants, the Nordic Fertility Society has tried to force companies to reveal this information without success. The medical staff we talked with told us that in the early days of clinical IVF work, they had been able to make their own mediums as laboratory craftwork; now they were dependent on standard mediums provided by large companies, because EU regulations only permitted CE-tested and certified mediums, which only big companies were able to produce.

As a result, the choice of a medium for one’s IVF laboratory is made without full information.

We are dependent on the commercial growth mediums [rather than being able to make them in-house]. The one thing I have always wished for is that at some point the producers of the mediums should be supervised, just like IVF medications are controlled. They are not controlled. They still have these product secrets about what gets added into the mediums. I really hope there will be [a change] because we put embryos in them. And these firms can add some preparation that will make more beautiful embryos, embryos that develop faster. What are the long-term effects? Overall, I think we should pay attention not so much to what kind of results the clinic has, how many pregnancies we manage to induce, but think more about the effects [of embryo culture and IVF] on the individual’s health and the health of the children and health later in life. This is something that has always worried me personally. … What is being done when, for instance, some growth hormone is added into some mediums. No one knows what effects it has. And then it may be a commercial secret, so we don’t even know what exactly is in them. (embryologist 4, clinic C)

This embryologist sees the fact that commercial establishments often do not disclose the exact composition (see Landecker, 2016) of their mediums as ethically dubious and as needing to be controlled in the way that IVF medications are controlled. There is suspicion that pharmaceutical companies add chemicals to the mediums to make the embryos morphologically beautiful and enhance their developmental performance – to improve the embryo vitality as assessed in the grading models that ultimately lead to the choice of an embryo for transfer. There is concern about the implications of this for the long-term health of embryos and children.

This concern seems legitimate, as can be seen from a story one embryologist told us about a sudden decrease in successful IVFs at her clinic: ‘The medium manufacturer had changed something. That was revealed, but first they said they had made no changes, but then there came news from other places as well that there have been problems. Then they confessed’ (embryologist 1, clinic E). It seems that the absence of information from the pharmaceutical industry, which is explained away in terms of market advantage, not only hinders the possibility of producing and knowing embryonic human life, but is also a liability for that life *in vitro*.

Embryologists are aware that culture mediums only partially and artificially mimic uterine surroundings, turning optimization into an uncertain and risky affair. In contrast to the historical institutional goal of neutralizing variability and making environments inert, the medium is reconceptualized from a mere adequate background condition to a constitutive factor in the making and knowing of human life forms. This scientific approach to epigenetics necessitates attention not just to the medium’s biomaterials as interactive agents, but also to the social, cultural, economic and political constitution of the material setting (Alder, 2013; Landecker, 2016: 149).

The industrial setting of culture mediums for IVF also means that individual clinics and chains do not share knowledge about their materials and practices for business reasons. This caused frustration among the embryologists in our study:

Everyone does [embryo culture] in their own different ways [refers to clinics] and it annoys me enormously that in this business results are not comparable, really. You just think that, oh, so you are transferring that sort of an embryo, I wonder what it would look like at our place [if we were to choose same embryo]. It is an established fact that embryos grow faster in some growth mediums. If you think about that, when you examine some other [embryo], it looks at this moment like that. Can you trust anything? If everything has an effect, it makes you feel awful, like this is not working. There is no absolute [truth] when the reality is that it varies. … But then we think, shall we use a different medium, but how do we know [how they work]? Based on one medium we could say this is a good embryo and based on another we could say this is too fast and this is bad. (embryologist 2, clinic C)

This embryologist is very aware that the embryos as known are the product of the growth medium. This knowledge also has consequences: The embryos are graded differently and different kinds of embryo are transferred into the intended mothers’ wombs at different clinics. However, as with the large pharmaceutical corporations, it is not in the local private clinics’ market interest to share their data: They prefer to keep their best practice, acquired through time-consuming laboratory tinkering and trial and error, to themselves. There is economic value in knowledge that is not shared. In the commercial industry setting, economic value wins out over the ethical value of openness.

The valuable knowledge here is not just knowledge of the type of medium used, but also local know-how (see Levin and Leonelli, 2017). Making a standard medium work is not automatic. Rather, it is an accomplishment arrived at in the laboratory through careful, non-standard craftwork. The embryologists in our study seemed reluctant to change the medium brand they were using, which they had found to *work* by doing and experiencing laboratory work.

We don’t change them often. We start using [new mediums] if … it is more usable, like easier to use. (embryologist 1, clinic E)

Sometimes we test new mediums from a different company. Usually I think there is no difference. These tools are often [chosen] according to what fits your hand the best and which one helps you work better [with the embryos]. (embryologist 2, clinic D)

The industrially produced (and not fully known) mediums are tested on embryos to see how they work in practice, to see how the embryos adapt to them. Via their own testing, the clinics achieve higher pregnancy rates and improve the practicalities of everyday laboratory work. Making mediums work by testing involves not just the context of production of the medium itself, but also the individual clinic and its hands-on labor. However, in clinical IVF settings, the professionals are not able to isolate the substances that are necessary or harmful for continued life because the composition of the mediums is not known. The professionals also don’t know the (probably) innumerable empirical tinkerings and tests that take place in pharmaceutical industry laboratories before a medium is released to market, even though those tests are part of the co-constitution of future embryos, inseparable from their *in vitro* milieu. As one embryologist summed up, ‘we just have to trust that they have been properly tested’ (embryologist 2, clinic D).

For the same reason of non-disclosure, many embryologists prefer to choose all their mediums from the same provider – the embryo growth medium, freezing medium, thawing medium and so on – as if they will fit together better. For pharmaceutical companies, this purchasing logic means that selling one medium equals selling a whole product line.

We choose the manufacturers according to experience and then we prefer to take all of the mediums from the same manufacturer because they work together well …. It is difficult to change them because they have different substances in them and we want the whole family then, so to speak. (embryologist 4, clinic C)

One might easily think that standard medium products bought from big transnational pharmaceutical providers would make business easier for local clinics, as they would not have to invest time and resources in making their own mediums. Although it may take a period of careful craftwork to get purchased mediums to work with the embryos, the clinics should be able to stick with the same products for a long time thereafter, without having to repeat the process. This industrial logic of cost-effectiveness is certainly something the big pharmaceutical companies promote. However, in some respects, both economic and ethical value gets lost in these forced dealings with pharmaceutical corporations, since information on embryo epigenetics is not shared.

It is no surprise, then, that when talking about mediums with the professionals today, they bring into the conversations the early days of maverick small businesses, when they used to craft their own. Such practice appears as a (politically) more desirable practice, with a different distribution of knowledge.

## Conclusion: The bio-economies of knowing embryonic life

In this article, we have explored embryo culture in clinical IVF laboratories as a knowledge production practice and process that ultimately aims to know and select the most viable embryos for transfer. We have discussed the ways in which incubator technologies (with and without TLS add-ons) and embryo culture mediums enable and disable knowledge about embryo morphology, embryogenesis and epigenetics. Our results regarding knowledge production are a contribution to discussions of craftwork and standardization in bioindustrialisation, which have hitherto mostly been discussed in the context of research laboratories rather than clinical laboratories (however, see e.g. Pavone and Arias, 2012; Van de Wiel, 2019).

Prior research suggests that transnational pharmaceutical and biotechnological giants rule the market for the commercialized products used in IVF; they also aggressively promote standardization and automation in clinics (Franklin, 2013; Global Fertility Alliance, 2018; Van de Wiel, 2019). Standardization and automation can be also seen as requiring clinics to scale up and branch out into new areas, geographically and otherwise (Franklin, 2013; see also Meskus, 2018). Despite this interest in capitalizing on the expanding fertility market and life science-led expansions more broadly, automation and standardization are only possible up to a point. This means that the future of bioindustrialization depends on the successful financializaton and commercialization of a mixture of human effort (patients and medics, in our case), standards, machinery and the labor of living cells, as Franklin (2013) points out. Our study offers a novel view into a few such possible mixtures in IVF laboratories.

Our study confirms previous findings (Foley and Whitaker, 2012; Muniesa et al., 2017; Petersen and Krisjansen, 2015) that there is a persistent gap between the enthusiastic market expectations and fuss around new biotechnologies and their actual industrial success, including those in clinical laboratories. TLS is a good example of this. While it is celebrated as a revolutionary technology that makes an epistemic break in the practices of knowing embryo viability by enabling staff to predict development times (see also Van de Wiel, 2018, 2019), TLS turns out to be not so practical in clinical practice after all, or gets used in non-standard and non-automated craft ways. This is not just because of the uncertainties and unpredictabilities of the performance of living material, but also because local economic interests do not always coincide with the larger industrial interests in automation and do not exclude concern over patients’ finances.

Furthermore, while it is perhaps interesting for research, much of the detailed knowledge about morphological features and embryogenesis enabled by TLS seems to make no difference in clinical work and the predictive component – which is based on algorithmic analyses of historical data sets and ideally allows the automation of knowledge production – is regarded as unnecessary and lacking. The craft of embryo culture also has value in itself: The valued goal is to keep embryos perceived as optimal for transfer alive and well *in vitro* until the blastocyst phase.

The second technology of much embryo culture – embryo culture mediums – enables and enacts knowledge about not just embryogenesis, but also epigenetics. However, because of supranational and national safety requirements, pharmaceutical giants have a monopoly over the mediums’ distribution and they refuse to share information about their composition. This is not uncommon in commercial settings (Landecker, 2007, 2016). In order to ‘get the job done’, clinicians simply need to trust the standards (see Timmermans, 2015: 80). However, professionals call for control from regulators to force this information into the open in the name of liability for human life.

Arguably, in our view, this lack of regulation results from the historical understanding of mediums as mere uterine-like backgrounds, rather than as co-constitutive artificial agents in embryo culture, which is how mediums are perceived in epigenetics. This suggests that political attention to the issue is needed. After all, the ultimate ‘product’ of the laboratory practices in IVF is a human person and the practices might bear consequences for following generations.

While commercial clinical establishments are not under the same pressure as scientific research to share knowledge/data (Birch, 2017; Levin and Leonelli, 2017), they are embedded in multiple exchanges and expectations. Hilgartner (2012) has argued that data-sharing follows a ‘dialectic of revelation and concealment’ through which knowledge is strategically made available and unavailable. In the case of IVF clinics, what happens in a situation where there is no open information about standard medium composition is that clinics make standard commercial mediums work in their own laboratories, which involves bioassay experimentation with embryos to see how they adapt to it. This experimentation is time-, resource- and labor-intensive and involves and develops local know-how. Thus, it comes as no surprise that the clinics, in turn, do not want to share this valuable know-how with other clinics or the larger industry: It is an achievement they want to capitalize on themselves. However, this results in less knowledge about the mediums’ epigenetic effects, further consolidating the bioindustry market, since embryologists feel that it is risky not to buy a whole line of laboratory pharmaceutics from the same provider.

The non-disclosure of milieu information, which affect embryonic life, causes frustration among professionals, who feel responsible toward embryos, future babies and intended parents. They are emotionally invested in the craftwork, and as part of this, disclosure/non-disclosure involves ethical as well as market value. Laboratory work is about both instrumentality and care, which, as Meskus (2018) argues, are mutually inclusive and interdependent when one seeks to make biomaterial work in the ways hoped and planned in the age of bioindustrialization (see also Adamson, 2010; Davies and Horst, 2015).

Can we then say that there are multiple bio-economies being drafted in clinical practices, albeit not very explicitly all the time? These also include bio-economies where mass production is not the ultimate goal – that is, where the primary obstacles to automation and standardization are not biological uniqueness, uncertainty or unpredictability. Rather, we are talking about practices where craft kicks back. Such practices can also be seen as models for reproductive care, drawing on a value system that disdains the mass industrial approach.
